# Rapidly *In Situ* Forming Platelet-Rich Plasma Gel Enhances Angiogenic Responses and Augments Early Wound Healing after Open Abdomen

**DOI:** 10.1155/2013/926764

**Published:** 2013-12-08

**Authors:** Bo Zhou, Jianan Ren, Chao Ding, Yin Wu, Dong Hu, Guosheng Gu, Jieshou Li

**Affiliations:** Department of Surgery, Jinling Hospital, Medical School of Nanjing University, 305 Zhongshan East Road, Nanjing 210002, China

## Abstract

*Objective.* The purposes of our present study were to evaluate the potential of platelet-rich plasma gel to enhance granulation tissue formation after open abdomen and to examine whether the effect was attributable to stimulating rapid neovascularization. *Methods.* Twenty-four rats underwent colon ascendens stent peritonitis surgery to induce sepsis, followed by intraperitoneal injection of nitrogen to create intra-abdominal hypertension. Four hours later, laparotomies were performed. The rats were randomized into three groups (*n* = 8 for each group): control, platelet-poor plasma (PPP), and platelet-rich plasma (PRP) groups. One week after the treatment, granulation tissue formation and angiogenesis were evaluated by histological and laser Doppler analysis. *Results.* The resultant platelet count in platelet-rich plasma was higher than that of PPP. The concentrations of platelet-derived growth factor BB, transforming growth factor **β**-1, and vascular endothelial growth factor in PRP were significantly higher when compared with that of PPP. Myofibroblast count, granulation tissue thickness, vessel numbers, and blood perfusion were increased in PRP group, followed by PPP group, with control being the least. *Conclusion.* Rapidly *in situ* forming platelet-rich plasma gel promoted remarkable neovascularization and early wound healing after open abdomen and may lead to novel and effective treatments for open abdominal wounds.

## 1. Background

Leaving the abdomen open (also laparotomy) has been widely used in a variety of surgical emergencies for its potential benefit to severely injured patients, involving abdominal compartment syndrome, intra-abdominal sepsis, trauma, and combat casualties [1–3]. Damage control laparotomy usually achieves a higher rate of primary fascial closure, but the management of the infected open abdomen (OA) poses substantial challenges to surgeons [4–6], and often the patient is left with an “open abdomen” until sufficient granulation of the intestinal convolutions followed by skin grafting. Therefore, promoting early stage granulation tissue formation is indispensable for those patients if primary fascial closure cannot be achieved.

The healing process of open abdominal wounds involves a complex and dynamic series of overlapping phases [7], in which recruitment of repairing cells, growth factors, and scaffold are critical to reconstituting tissue integrity. Platelet-rich plasma (PRP) gel, structurally similar to the natural fibrin clot [8], can be used as scaffold for cells infiltration and assembly of vascular networks. Also, PRP gel can be used to deliver high quantities of key growth factors, such as platelet-derived growth factor AB (PDGF-BB), transforming growth factor *β*-1 (TGF*β*-1), and vascular endothelial growth factor (VEGF), and recruit repairing cells to the site of tissue damage [9, 10], which are essential to natural wound healing. In fact, the topical use of platelet-rich plasma gel has been advocated for numerous clinical indications [11–13]. These observations and inferences led to the hypothesis that PRP gel supplementation would accelerate the open abdominal wound healing. However, no research has examined the potential of PRP gel as part of treatment to promote wound healing after the open abdomen.

In this study, we used rapidly *in situ* forming scaffolds via platelet-rich plasma and platelet-poor plasma (PPP) in conjunction with a clotting agent (typically bovine thrombin) to treat open abdominal wounds. Our aim was to evaluate if treating OA wounds with PRP gel would significantly enhance the OA wound-healing process and reduce the time required to achieve adequate granulation tissue formation in order to undergo skin grafting, and to examine whether the effect was attributable to stimulating rapid neovascularization.

## 2. Materials and Methods

### 2.1. Experimental Animals

Forty-eight adult male Sprague-Dawley rats (180–250 g, Jinling Hospital, Nanjing, China) were used for the present experiments. The animals were maintained in a controlled environment (21 ± 2°C, 50–60% humidity, 12-hour light-dark cycle, and lights on at 6 am) and allowed free access to food and water. All the animal care and experimental protocols were reviewed and approved by Animal Investigation Ethics Committee of Jinling Hospital.

### 2.2. Preparation of Platelet-Rich Plasma

PRP was prepared by enriching whole blood platelet concentration using a two-step centrifugation procedure. Ten milliliters of whole blood was drawn from healthy rat through cardiac puncture into prechilled tubes containing ACD-A at a blood/ACD-A ratio of 9 : 1. Subsequently, each blood sample was centrifuged at 400 ×g for 10 min to obtain the three typical layers: red blood cells at the bottom, a “buffy coat” layer in between, and acellular plasma in the supernatant. Using a sterile pipette, the upper layer was transferred to another neutral tube along with the buffy coat and recentrifuged at 800 ×g for 10 min. About 2 mL of PRP was omitted from the bottom of the tube and about 2 mL of PPP was collected in the supernatant to yield the final PRP and PPP product, respectively. The final platelet concentrations in whole blood, PPP, and PRP were analyzed in an automatic counter. Samples of PRP and PPP were frozen at −80°C and then thawed in cold water in order to lyse the platelets. The concentrations of VEGF, TGF*β*-1, and PDGF-BB in whole blood, PPP, and PRP were measured by enzyme-linked immunosorbent assay (ELISA) according to the manufacturer's instructions.

### 2.3. Surgical Procedures and PRP Gel *In Situ*

All rats were fasted overnight and anesthetized by intraperitoneal injections of a ketamine (50 mg/kg body weight) and xylazine (5 mg/kg body weight) mixture. Under aseptic conditions, the colon ascendens stent peritonitis (CASP) procedure was performed to create a continuous intra-abdominal sepsis [14]. In brief, a 3 mm long venous indwelling cannula (14G, Venflon, Ohmeda, Sweden) was inserted and fixated into the colon ascendens, approximately 1.5 cm distal to the ileocecal valve, at the antimesenteric site. By careful palpation of the cecum, the cannula was filled with feces. Subsequent to repositioning of the colon ascendens and fluid substitution using 2 mL sterile saline solution, the layers of the abdomen (muscular and skin) were sutured (5/0 Ethicon). Then, a silicone catheter (outer diameter 0.8 mm) was inserted into the abdominal cavity for nitrogen gas insufflation, maintaining 20 mm Hg of abdominal pressure [15]. After 4 hours, we opened the suture of the abdominal wall, closed the defect in the colon with single inverting sutures (5/0), and flushed the abdominal cavity with 10 mL of saline solution. Then we removed full-thickness abdominal wall, thereby creating a 2 cm × 3 cm defect.

**Figure 1:**
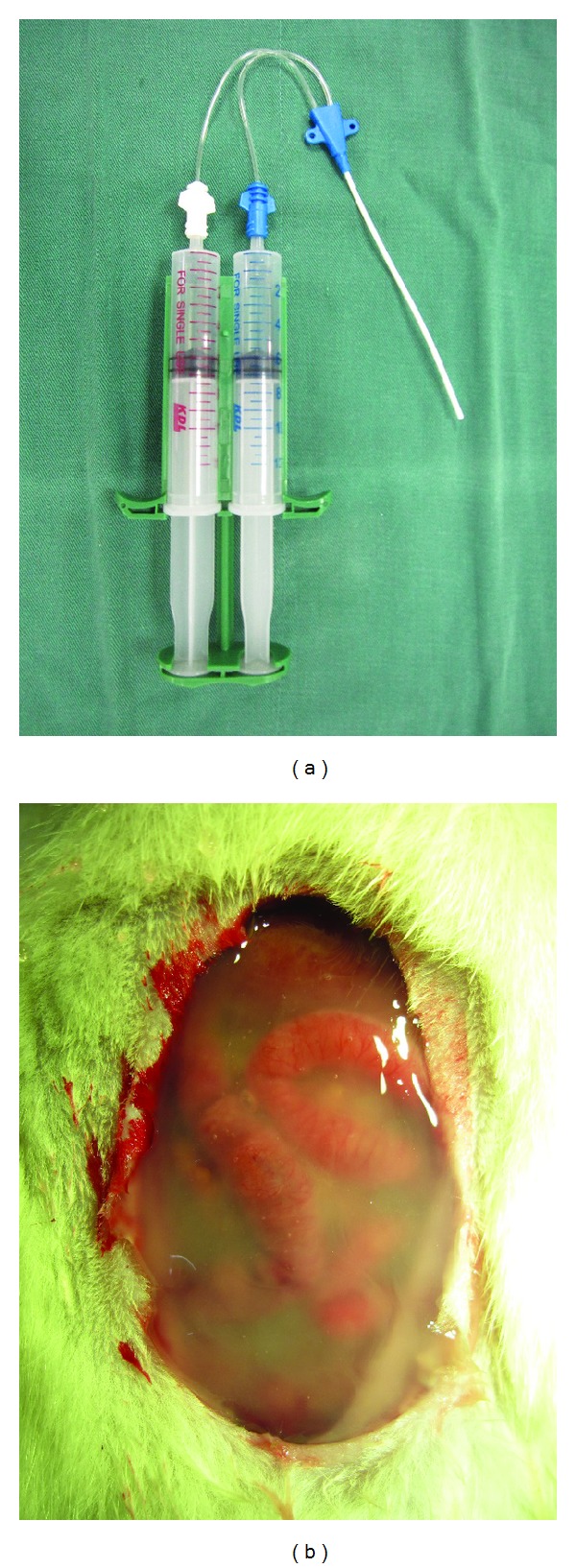
(a) A double-syringe arrangement for dispensing PRP gel and (b) topical application of PRP gel to the open abdominal wound.

After that, the animals were randomly divided into three groups: the PRP group, the PPP group, and the control group, with eight rats in each group. PRP or PPP gel was administered as a two-component system: the prepared PRP or PPP as one component and a thrombin/Ca^+2^ composition as the other. The system used a double-syringe arrangement wherein the two components were mixed *in situ* immediately prior to dispensing to open abdominal wound (Figure 1(a)). In the PRP group and the PPP group, the wound was covered with the same size of PRP or PPP gel (Figure 1(b), both with thickness of approximately 0.3 cm), respectively, and then layered with DuoDerm, an extra thin dressing, to enable gel placement. Finally, the abdomen was temporarily closed using aseptic polypropylene mesh (Budd Company, Troy, MI). In the control group, the wounds were covered only with the mesh.

### 2.4. Histology

The granulation tissue together with underlying bowel loops was collected at day 7 and fixed with 10% neutral formaldehyde, followed by dehydration in graded ethanol (70% to 100%), embedding in paraffin, serially section using a microtome (5 *μ*m), and subsequent staining with either hematoxylin and eosin (H&E) or immunohistochemistry for CD31 and *α*-SMA (Abcam, Cambridge, MA).

### 2.5. Laser Doppler Analysis

Blood perfusion in wound areas was measured with a laser speckle contrast imaging (LSCI, PeriCam PSI System, Perimed, Sweden) for 2 min, to ensure temporal stability between measurements. The system uses a divergent laser beam with a wavelength of 785 nm. The spatial resolution of the perfusion image is 0.2 mm/pixel at a measurement distance of 12 cm. The image size was set to correspond to 2 cm × 3 cm and the image acquisition rate was set to 3 images/s. Data were digitized and stored in a computer, and mean perfusion levels in regions of the image were analyzed offline with signal processing software (PimSoft 1.4, Perimed AB, Sweden). All LSCI blood perfusion measurements are presented in laser speckle perfusion units (LSPU).

### 2.6. Statistics

Data are presented as means ± SEM unless otherwise noted. All measurements were preformed from at least six different slides or rats, with multiple readings for each data point. Continuous variables were analyzed by one-way ANOVA as appropriate. A repeated measure of analysis of variance with a post hoc LSD test was used when comparing more than two variables. All statistical analyses were performed with IBM SPSS Statistics 18 (SPSS Inc., Chicago, IL, USA) and *P* values < 0.05 were considered statistically significant.

## 3. Results

### 3.1. Platelet Concentration in Whole Blood, PPP, and PRP

The platelet count in the whole blood, PPP, and PRP had a mean value of 0.84 ± 0.16, 0.05 ± 0.01, and 2.34 ± 0.46 × 10^9^/L, respectively. The PRP contained about 2.47 times the number of platelets found in the whole blood sample, and the PPP contains only 6% of the original platelet count.

### 3.2. Growth Factors in PPP and PRP

**Figure 2:**
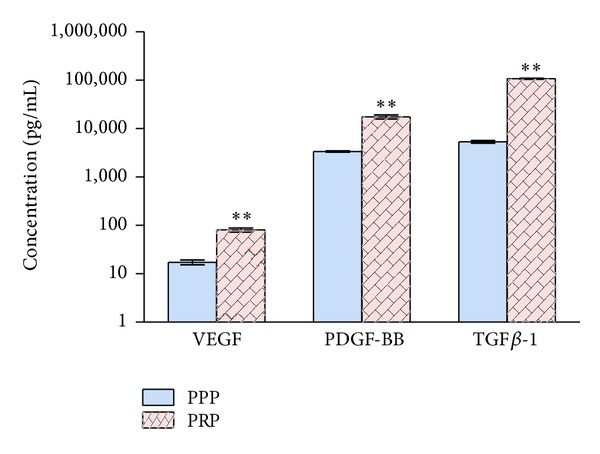
Quantification of growth factors (pg/mL) in PRP and PPP. All samples were freeze-thawed to lyse the platelets prior to measurement. **P < 0.01.

As shown in Figure 2, various GFs were tested and different concentrations of GFs were obtained in PPP and PRP. For VEGF, 17 ± 5 and 81 ± 22 pg/mL were, respectively, determined in PPP and PRP. For TGF*β*-1, 5368 ± 954 and 107636 ± 7837 pg/mL were, respectively, determined in PPP and PRP. For PDGF-BB, 3380 ± 353 and 17517 ± 4688 pg/mL were, respectively, determined in PPP and PRP. All values of these growth factors in PRP were significantly higher than those of PPP (*P* < 0.05).

### 3.3. PRP Gel Promotes Early Wound-Healing Process

Histological analysis revealed that, compared to control group, PRP gel yielded improved healing response, which showed a much more rapid cellular accumulation and matrix deposition. To accurately quantitate the amount of new tissue, we chose three other independent measures of tissue formation—granulation tissue thickness, myofibroblasts count, and vessel numbers and diameter.

**Figure 3:**
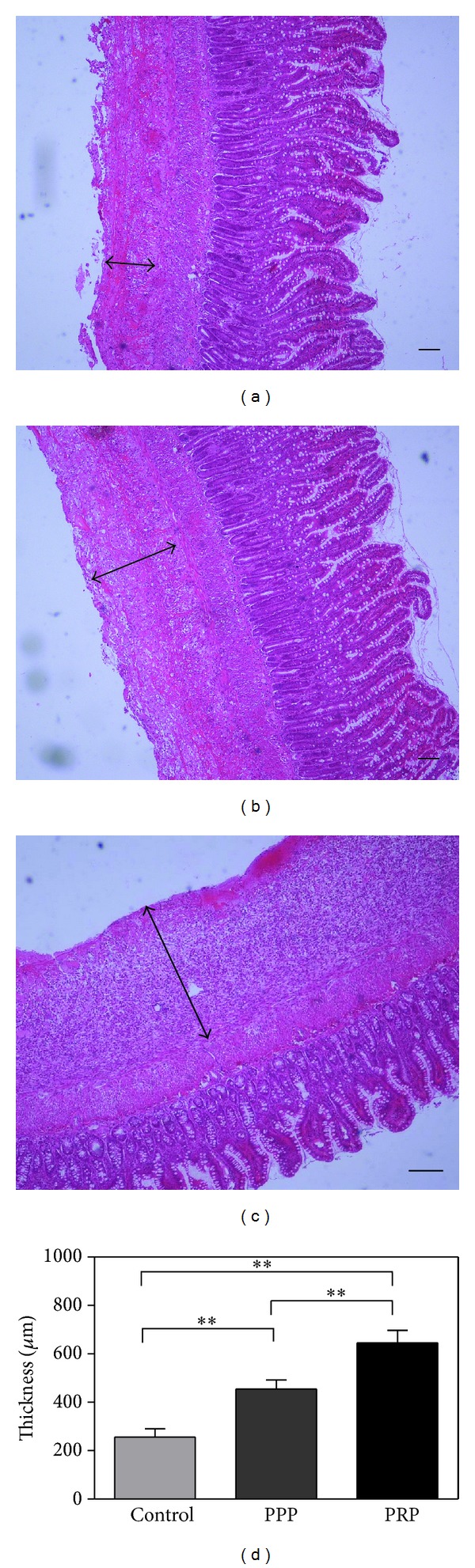
Thickness of the granulation tissue at one week after treatment in control (a), PPP (b), and PRP (c) group. (d) The statistical analysis of the granulation thickness in the three groups. Scale bar indicates 100 *μ*m. **P < 0.01.

**Figure 4:**
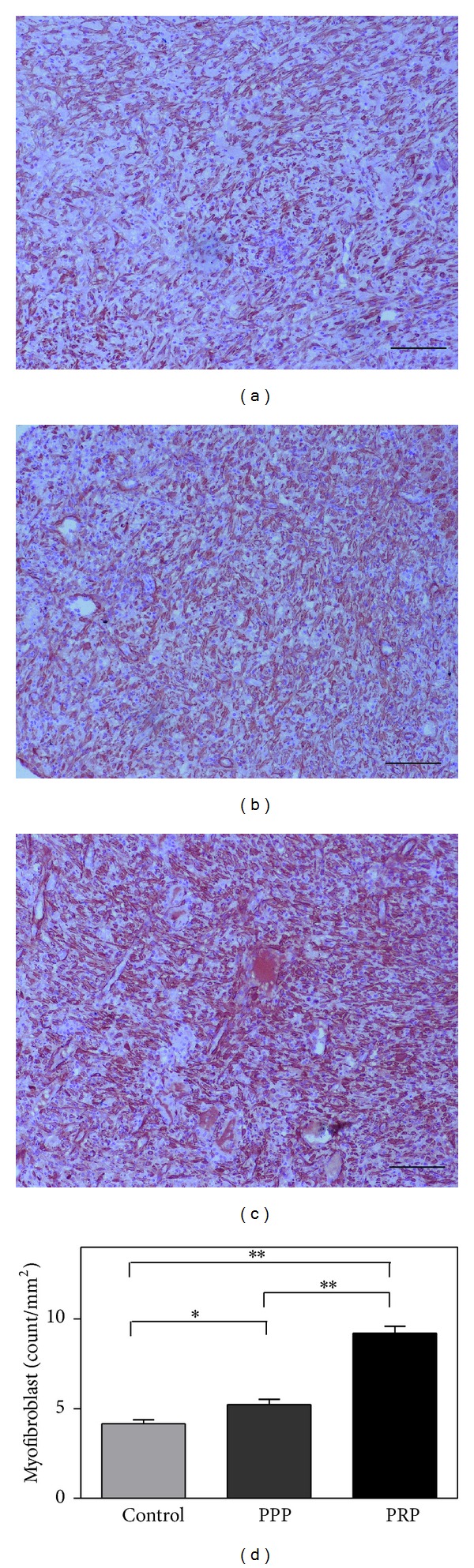
*α*-SMA immunostaining analysis of granulation tissue myofibroblast at one week after treatment in control (a), PPP (b), and PRP (c) group. (d) The statistical analysis of the percentage of *α*-SMA positive area in the three groups. Scale bar indicates 100 *μ*m. *P < 0.05; **P < 0.01.

**Figure 5:**
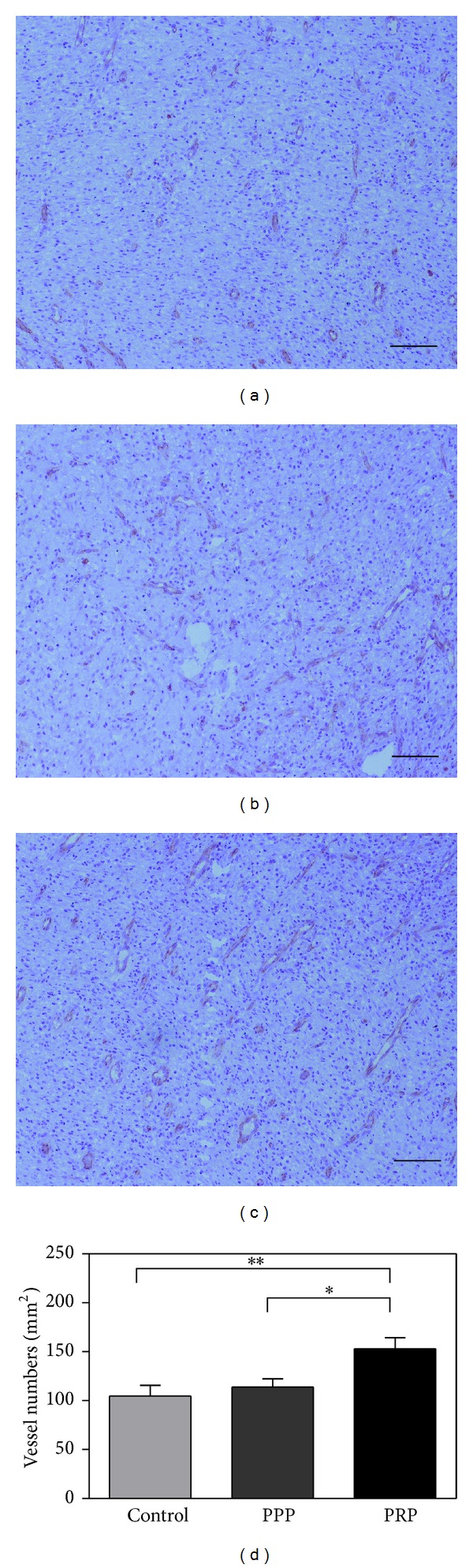
CD31 immunostaining analysis of granulation tissue capillaries at one week after treatment in control (a), PPP (b), and PRP (c) group. (d) The statistical analysis of the percentage of CD31 positive area in the three groups. Scale bar indicates 100 *μ*m. *P < 0.05, **P < 0.01.

PRP gel treatments induced significant 1.4- and 2.5-fold increase, respectively, in granulation tissue thickness compared with the PPP and control group (Figure 3). SMA staining showed that PRP group exhibited a significant increase in myofibroblast positive area compared with PPP and control group (Figure 4). Moreover, increased tissue vascularization in response to treatment with PRP gel was observed on day 7, as evidenced by positive CD31 staining (Figure 5), when compared with PPP and control group.

### 3.4. PRP Gel Promotes Angiogenic Response

**Figure 6:**
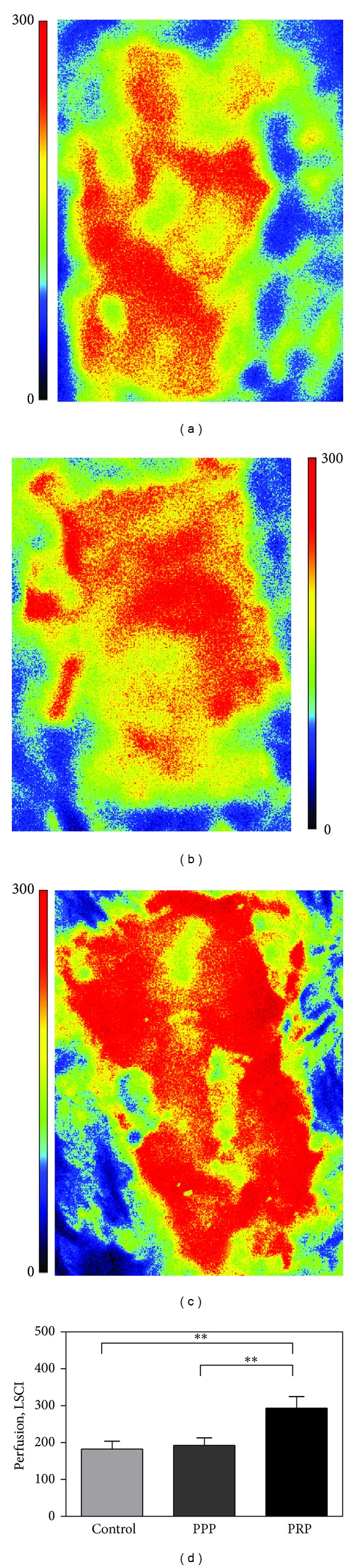
Laser speckle contrast imaging of open abdominal wound at one week after treatment in control (a), PPP (b), and PRP (c) group. (d) The statistical analysis of blood perfusion in the three groups. Scale bar indicates 100 *μ*m. **P < 0.01.

To better determine the functionality of the developing vasculature, we analyzed wounds on day 7, using laser Doppler to assess blood perfusion in the wound area. We found that PRP gel induced more blood flow to the wound area than did the PPP scaffold and the wound covered with only mesh (Figure 6). For example, the blood perfusion with PRP gel was 293 LSPU, whereas the blood perfusion was only 193 and 183 LSPU for PPP and control group, respectively. There was no significant difference between PRP gel-treated wounds and controls.

## 4. Discussion

In this study, we have demonstrated the benefits of topical platelet-rich plasma gel for the treatment of open abdominal wounds. We have shown that wounds treated with PRP gel exhibited faster healing rates and adequate granulation tissue formation when compared to wounds treated with mesh alone, subsequently reducing the time it takes to undergo skin graft. In addition, topical use of PRP gel has been shown to enhance angiogenesis in the early stage of the repair process after open abdomen and subsequently to promote wound healing.

Appreciation for the potential complications of open abdominal wound has continued to evolve. Accordingly, various surgical techniques and nonanatomic coverage alternatives for early restoration of abdominal domain after OA have been proposed. Generally, the optimal goal of early management is to facilitate early closure (within the first 7 days) and prevent delayed complications. However, in some cases, especially in an infected abdomen, primary abdominal fascial closure is not possible secondary to ongoing visceral edema and depleted fascia edges due to inflammation and lateral retraction. In these cases, the patient is left with an “open abdomen” until sufficient granulation. Thus, promoting early granulation tissue formation is necessary to prevent complications.

The use of blood-derived biomaterials to seal wounds and accelerate healing began with the use of fibrin glues in the early 1970s, which comprised a highly concentrated fibrinogen (polymerization induced by thrombin and calcium) [16, 17]. It was then first introduced by Girard et al. in 2002 in patients with open abdomen to promote the healing of intestinal fistula [18]. Even now, ten years later, their use for treatment of open abdominal wounds remains very limited due to the cost and the complexity in production [19]. Consequently, the use of platelet concentrates to stimulate healing and replace fibrin glues, as first described by Whitman et al. [20], has increased its popularity during the last decade in many clinical conditions [12, 21, 22]. However, to the best of our knowledge there is no previous study to evaluate the effect PRP gel on wound healing after open abdomen and its mechanism.

In general, wound healing has three classic stages: the inflammatory, proliferative, and remodeling stages [7]. The early stage of wound repair begins with hemostasis and formation of the platelet plug, followed by a fibrin matrix, which becomes the scaffold for infiltrating cells involved in angiogenesis and tissue repair. However, the following characteristics make infected open abdominal wound different from the healing process we have described above: infected open abdominal wounds, similar to chromic wounds, are thought to have increased proteases and decreased protease inhibitors. Thus these could reduce the ability of forming new tissue. PRP gel, structurally similar to the natural fibrin clot [8], cannot only provide structural support for growing cells but also regulate the production of matrix metalloproteinase [23], thereby accelerating the healing process.

Furthermore, platelet concentrates contain many powerful mitogenic and chemotactic growth factors, which regulate key processes involved in tissue repair, including cell proliferation, chemotaxis, migration, cellular differentiation, and extracellular matrix synthesis [10, 24]. PDGF, bFGF, TGF*β*, IGF, and EGF are chemotactic for fibroblasts [25, 26]. Both VEGF and bFGF induce a pro-angiogenic phenotype in cultured endothelial cell [27]. Bone marrow stromal cells, which are recruitable during the tissue-repair process, are upregulated by PRP as well [28]. Several growth factors, such as VEGF, PDGF, and bFGF, become intricately involved in angiogenesis [29, 30]. All of the above-mentioned growth factors are released by PRP gel. Also, bFGF and TGF*β* concentrations are downregulated in chronic wounds and are much lower than those reported from acute wounds [31]. In addition, by using the PRP gel as a growth factor source to dress the wound, hence, *in vivo* treatment, their local availability for promoting healing should be guaranteed by control release of growth factors.

In addition, the observed effect may be partially due to its antimicrobial activity. The roles that platelets in PRP play in host defense mechanism at the wound site have been demonstrated by previous studies [32, 33]. Both pure platelet-rich plasma and platelet-leukocyte gel inhibited *Enterococcus faecalis*, *Candida albicans*, *Streptococcus agalactiae*, and *Streptococcus oralis*. This might represent a valuable property regarding the enhancement of wound healing after open abdomen.

We propose PRP gel for use in clinical practice. It can be accessible to most physicians, whether in metropolitan areas or in those areas with hospital facilities. PRP gel is easy to prepare from only 20–40 mL autologous blood of the patient and is of relatively low cost. All available PRP techniques share common principles: blood is collected with an anticoagulant just before use and is immediately centrifuged twice. This time is variable but is always completed within no more than an hour. Also, application of autologous PRP gel to the wound site is technically easy and could be used as a conventional nonoperative therapy. The obtained platelet concentrate, together with thrombin and calcium chloride, is placed separately in a double-syringe system with a distal mixing device.

A variety of techniques, with the goal of either achieving definitive primary fascial closure (DPC) or restoring abdominal domain, are now available after open abdomen, including Bogota bag, the Wittmann Patch, synthetic mesh, VAC device, or combinations of various approaches [6, 34, 35]. To date, however, there are no data suggesting that one is particularly better than the other in facilitating DPC [4]. A UK national study has reported that NPWT was associated with a 27% reduction (only 44.9%) in delayed primary closure rate in patients who had their abdomen left open for the management of sepsis [5]. The low rate of delayed primary closure suggests that these techniques, including NPWT, may be used as definitive treatment in the setting of intra-abdominal sepsis, with the use of biomaterials, such as PRP gel, to obtain visceral coverage until the wound can undergo skin grafting. Although the approach of combining synthetic materials and biologically derived components is attractive in that it can promote wound healing while preserving the mechanical properties, additional study is required to determine the optimal algorithm for the management of open abdomen after abdominal sepsis.

As a preliminary study, several limitations need to be addressed. First, a potential weakness of our approach is that allogeneic instead of autologous PRP was used. Originally, PRP is defined as an autologous concentration of platelets in a small volume of plasma [9]. In experimental model such as rats, the blood volume is too small to produce autologous PRP, thus necessitating the use of donor blood for preparation of PRP. According to Marx [9], the use of donor animal blood platelets conveys a risk of imparting an overt immune reaction, which may lead to false-negative results. However, the latest study could remove such concerns because of the demonstration of its positive effects [36]. Second, this study just focuses on the early process of wound healing, but has not evaluated the progress in subsequent wound healing further effect on organ function, skin graft, and associated complications. In the next stage, we need to examine the consequences of the observed results.

In conclusion, rapidly *in situ* forming platelet-rich plasma gel promoted remarkable neovascularization and wound healing in the early stage after open abdomen. These results encourage the further clinical study of the technique and may lead to novel and effective treatments for open abdominal wounds.

## References

[1] Morrison JJ, Poon H, Garner J (2012). Nontherapeutic laparotomy in combat casualties. *The Journal of Trauma and Acute Care Surgery*.

[2] Carr JA (2013). Abdominal compartment syndrome: a decade of progress. *Journal of the American College of Surgeons*.

[3] Schecter WP, Ivatury RR, Rotondo MF, Hirshberg A (2006). Open abdomen after trauma and abdominal sepsis: a strategy for management. *Journal of the American College of Surgeons*.

[4] DuBose JJ, Scalea TM, Holcomb JB (2013). Open abdominal management after damage-control laparotomy for trauma: a prospective observational American Association for the Surgery of Trauma Multicenter Study. *The Journal of Trauma and Acute Care Surgery*.

[5] Carlson GL, Patrick H, Amin AI (2013). Management of the open abdomen: a national study of clinical outcome and safety of negative pressure wound therapy. *Annals of Surgery*.

[6] Turza KC, Campbell CA, Rosenberger LH (2012). Options for closure of the infected abdomen. *Surgical Infections*.

[7] Gurtner GC, Werner S, Barrandon Y, Longaker MT (2008). Wound repair and regeneration. *Nature*.

[8] Fernández-Barbero JE, Galindo-Moreno P, Ávila-Ortiz G, Caba O, Sánchez-Fernández E, Wang H-L (2006). Flow cytometric and morphological characterization of platelet-rich plasma gel. *Clinical Oral Implants Research*.

[9] Marx RE (2004). Platelet-rich plasma: evidence to support its use. *Journal of Oral and Maxillofacial Surgery*.

[10] Carter CA, Jolly DG, Worden CE, Hendren DG, Kane CJM (2003). Platelet-rich plasma gel promotes differentiation and regeneration during equine wound healing. *Experimental and Molecular Pathology*.

[11] Driver VR, Hanft J, Fylling CP (2006). A prospective, randomized, controlled trial of autologous platelet-rich plasma gel for the treatment of diabetic foot ulcers. *Ostomy Wound Management*.

[12] Kazakos K, Lyras DN, Verettas D, Tilkeridis K, Tryfonidis M (2009). The use of autologous PRP gel as an aid in the management of acute trauma wounds. *Injury*.

[13] Pallua N, Wolter T, Markowicz M (2010). Platelet-rich plasma in burns. *Burns*.

[14] Lustig MK, Bac VH, Pavlovic D (2007). Colon ascendens stent peritonitis—a model of sepsis adopted to the rat: physiological, microcirculatory and laboratory changes. *Shock*.

[15] Yuan Y, Ren J, Zhang W, Chen J, Li J (2011). The effect of different temporary abdominal closure materials on the growth of granulation tissue after the open abdomen. *The Journal of Trauma and Acute Care Surgery*.

[16] Dohan Ehrenfest DM, Rasmusson L, Albrektsson T (2009). Classification of platelet concentrates: from pure platelet-rich plasma (P-PRP) to leucocyte- and platelet-rich fibrin (L-PRF). *Trends in Biotechnology*.

[17] Matras H (1970). Effect of various fibrin preparations on reimplantations in the rat skin. *Österreichische Zeitschrift für Stomatologie*.

[18] Girard S, Sideman M, Spain DA (2002). A novel approach to the problem of intestinal fistulization arising in patients managed with open peritoneal cavities. *The American Journal of Surgery*.

[19] Gibble JW, Ness PM (1990). Fibrin glue: the perfect operative sealant?. *Transfusion*.

[20] Whitman DH, Berry RL, Green DM (1997). Platelet gel: an autologous alternative to fibrin glue with applications in oral and maxillofacial surgery. *Journal of Oral and Maxillofacial Surgery*.

[21] Hom DB, Linzie BM, Huang TC (2007). The healing effects of autologous platelet gel on acute human skin wounds. *Archives of Facial Plastic Surgery*.

[22] Vogrin M, Rupreht M, Dinevski D (2010). Effects of a platelet gel on early graft revascularization after anterior cruciate ligament reconstruction: a prospective, randomized, double-blind, clinical trial. *European Surgical Research*.

[23] Shin HS, Oh HY (2012). The effect of platelet-rich plasma on wounds of OLETF rats using expression of matrix metalloproteinase-2 and -9 mRNA. *Archives Plastic Surgery*.

[24] Kajikawa Y, Morihara T, Sakamoto H (2008). Platelet-rich plasma enhances the initial mobilization of circulation-derived cells for tendon healing. *Journal of Cellular Physiology*.

[25] Loots MA, Kenter SB, Au FL (2002). Fibroblasts derived from chronic diabetic ulcers differ in their response to stimulation with EGF, IGF-I, bFGF and PDGF-AB compared to controls. *European Journal of Cell Biology*.

[26] Seppa H, Grotendorst G, Seppa S (1982). Platelet-derived growth factor is chemotactic for fibroblasts. *Journal of Cell Biology*.

[27] Pintucci G, Froum S, Pinnell J (2002). Trophic effects of platelets on cultured endothelial cells are mediated by platelet-associated fibroblast growth factor-2 (FGF-2) and vascular endothelial growth factor (VEGF). *Thrombosis and Haemostasis*.

[28] Vaquero J, Otero L, Bonilla C (2013). Cell therapy with bone marrow stromal cells after intracerebral hemorrhage: impact of platelet-rich plasma scaffolds. *Cytotherapy*.

[29] Matsui M, Tabata Y (2012). Enhanced angiogenesis by multiple release of platelet-rich plasma contents and basic fibroblast growth factor from gelatin hydrogels. *Acta Biomaterialia*.

[30] Yuan Y, Ren J, Zhang W, Chen J, Li J (2011). The effect of different temporary abdominal closure materials on the growth of granulation tissue after the open abdomen. *The Journal of Trauma and Acute Care Surgery*.

[31] Cooper DM, Yu EZ, Hennessey P (1994). Determination of endogenous cytokines in chronic wounds. *Annals of Surgery*.

[32] Drago L, Bortolin M, Vassena C (2013). Antimicrobial activity of pure platelet-rich plasma against microorganisms isolated from oral cavity. *BMC Microbiology*.

[33] Moojen DJ, Everts PA, Schure RM (2008). Antimicrobial activity of platelet-leukocyte gel against staphylococcus aureus. *Journal of Orthopaedic Research*.

[34] van Hensbroek PB, Wind J, Dijkgraaf MG (2009). Temporary closure of the open abdomen: a systematic review on delayed primary fascial closure in patients with an open abdomen. *World Journal of Surgery*.

[35] Yuan Y, Ren J, Yuan K (2013). The modified sandwich-vacuum package for fascial closure of the open abdomen in septic patients with gastrointestinal fistula. *The Journal of Trauma and Acute Care Surgery*.

[36] Yamaguchi R, Terashima H, Yoneyama S, Tadano S, Ohkohchi N (2012). Effects of platelet-rich plasma on intestinal anastomotic healing in rats: PRP concentration is a key factor. *Journal of Surgical Research*.

